# Integrating Whole Blood Transcriptomic Collection Procedures Into the Current Anti-Doping Testing System, Including Long-Term Storage and Re-Testing of Anti-Doping Samples

**DOI:** 10.3389/fmolb.2021.728273

**Published:** 2021-10-26

**Authors:** Giscard Lima, Alexander Kolliari-Turner, Fernanda Rossell Malinsky, Fergus M. Guppy, Renan Paulo Martin, Guan Wang, Sven Christian Voss, Costas Georgakopoulos, Paolo Borrione, Fabio Pigozzi, Yannis Pitsiladis

**Affiliations:** ^1^ Department of Movement, Human and Health Sciences, University of Rome “Foro Italico”, Rome, Italy; ^2^ School of Sport and Health Sciences, University of Brighton, Eastbourne, United Kingdom; ^3^ Centre for Stress and Age Related Disease, University of Brighton, Brighton, United Kingdom; ^4^ School of Applied Sciences, University of Brighton, Brighton, United Kingdom; ^5^ Department of Biophysics, Federal University of Sao Paulo, Sao Paulo, Brazil; ^6^ McKusick-Nathans Department of Genetic Medicine, Johns Hopkins University School of Medicine, Baltimore, MD, United States; ^7^ Sport and Exercise Science and Sports Medicine Research and Enterprise Group, University of Brighton, Brighton, United Kingdom; ^8^ Anti-Doping Lab Qatar, Doping Analysis Lab, Doha, Qatar; ^9^ NADO Italia, National Antidoping Organization, Rome, Italy; ^10^ International Federation of Sports Medicine (FIMS), Lausanne, Switzerland

**Keywords:** RNA-Seq, frozen blood, RNA integrity, anti-doping, doping biomarkers, gene expression, microarray, Recombinant human erythropoietin

## Abstract

**Introduction:** Recombinant human erythropoietin (rHuEPO) administration studies involving transcriptomic approaches have demonstrated a gene expression signature that could aid blood doping detection. However, current anti-doping testing does not involve collecting whole blood into tubes with RNA preservative. This study investigated if whole blood in long-term storage and whole blood left over from standard hematological testing in short-term storage could be used for transcriptomic analysis despite lacking RNA preservation.

**Methods:** Whole blood samples were collected from twelve and fourteen healthy nonathletic males, for long-term and short-term storage experiments. Long-term storage involved whole blood collected into Tempus™ tubes and K_2_EDTA tubes and subjected to long-term (i.e., ‒80°C) storage and RNA extracted. Short-term storage involved whole blood collected into K_2_EDTA tubes and stored at 4°C for 6‒48 h and then incubated at room temperature for 1 and 2 h prior to addition of RNA preservative. RNA quantity, purity, and integrity were analyzed in addition to RNA-Seq using the MGI DNBSEQ-G400 on RNA from both the short- and long-term storage studies. Genes presenting a fold change (FC) of >1.1 or < ‒1.1 with *p* ≤ 0.05 for each comparison were considered differentially expressed. Microarray analysis using the Affymetrix GeneChip® Human Transcriptome 2.0 Array was additionally conducted on RNA from the short-term study with a false discovery ratio (FDR) of ≤0.05 and an FC of >1.1 or < ‒1.1 applied to identify differentially expressed genes.

**Results:** RNA quantity, purity, and integrity from whole blood subjected to short- and long-term storage were sufficient for gene expression analysis. Long-term storage: when comparing blood tubes with and without RNA preservation 4,058 transcripts (6% of coding and non-coding transcripts) were differentially expressed using microarray and 658 genes (3.4% of mapped genes) were differentially expressed using RNA-Seq. Short-term storage: mean RNA integrity and yield were not significantly different at any of the time points. RNA-Seq analysis revealed a very small number of differentially expressed genes (70 or 1.37% of mapped genes) when comparing samples stored between 6 and 48 h without RNA preservative. None of the genes previously identified in rHuEPO administration studies were differently expressed in either long- or short-term storage experiments.

**Conclusion:** RNA quantity, purity, and integrity were not significantly compromised from short- or long-term storage in blood storage tubes lacking RNA stabilization, indicating that transcriptomic analysis could be conducted using anti-doping samples collected or biobanked without RNA preservation.

## Introduction

The World Anti-Doping Agency (WADA) was created in 1999 and has been at the forefront of anti-doping ever since (Ljungqvist, 2017). Over the years, numerous testing methods and strategies have been developed to improve the efficiency of the anti-doping system with some success, thanks to advances in analytical technologies (Thevis et al., 2021). However, the efficiency of the current system remains inadequate as the number of true doping cases vastly exceeds those detected (de Hon et al., 2014; Faiss et al., 2020). In response to this situation and to take advantage of advances in analytical techniques over the last few decades, WADA introduced the capacity for anti-doping samples to be stored for the long term (initially 8 years, but currently 10 years) for retrospective re-analysis as new technologies and approaches emerge (Kuuranne and Saugy, 2016). Subsequently, the majority (74%) of Anti-Doping Rule Violations that have impacted medal-winning results at the Summer Olympic Games, from 1968 to 2012, have been identified retrospectively (Kolliari-Turner et al., 2021). Thereby, it has been argued that in addition to increasing pre-Olympic out-of-competition testing to avoid retrospective analysis to identify doping in the first place, long-term sample storage should continue and additionally incorporate novel and potentially complementary technologies/sample matrices (Kolliari-Turner et al., 2021).

The continued success of the storage and re-analysis program is predicated on the inclusion of new technologies as these become available. For example, next-generation sequencing and “omics”-based technologies are increasingly being used in clinical testing, scientific research, and disease diagnoses, including cancer, with immense success (Kaczor-Urbanowicz et al., 2017; Schmidt et al., 2021). It is anticipated that multi-omics approaches will, in time, be integrated into current biomarker assessment (International Olympic Committee, 2019). However, adoption of these omics-based methods for anti-doping purposes will require the appropriate storage of samples such as blood, urine, and saliva into specific preservatives (Beekman et al., 2009; Kaczor-Urbanowicz et al., 2017), which, until presently, was not being planned. This is despite the fact that the first studies emerged more than a decade ago, showing great potential for omics strategies and methods to improve anti-doping testing by discovering indirect doping biomarkers and analyzing biological changes induced by performance-enhancing drugs (PEDs) (Reichel, 2011; Saugy et al., 2011). These first studies of gene expression analysis in whole blood samples collected following recombinant human erythropoietin (rHuEPO) administration revealed a characteristic gene expression signature with important implications for rHuEPO doping detection (Durussel et al., 2016; Wang et al., 2017a). These studies, interpreted in the context of “omics” applications in the field of biomedical sciences (Karczewski and Snyder, 2018), reveal that it is only a matter of when, rather than if, these omics-based methods will become regularly used in anti-doping science, highlighting the importance for appropriate storage of samples for analysis using these methods when they become available. However, whole blood samples currently collected for anti-doping purposes (WADA, 2021a), such as K_2_EDTA tubes, do not contain RNA stabilizing reagents. Once collected, the whole blood samples are sealed in tamper-proof containers, stored upright in a refrigerated state, dispatched with a temperature data logger to the laboratory, and analyzed within 72 h from collection (WADA, 2021b). As such, whole blood samples are collected/stored in conditions that may not prevent RNA degradation, and therefore, it is unknown whether the degradation of RNA transcripts could confound subsequent transcriptomic analysis (Gallego Romero et al., 2014). Importantly, the implementation/integration of transcriptomic technologies into the current anti-doping system should use the same methodology as that used in the discovery and validation studies (Durussel et al., 2016; Wang et al., 2017a); when this is not possible, an adaptive protocol should be developed and validated. This study has two aims: 1) to study if whole blood collected into K_2_EDTA tubes and subjected to long-term storage (i.e., −80°C storage, to simulate biobanking of K_2_EDTA tubes) could impact transcriptomic analysis and 2) to investigate if whole blood collected into K_2_EDTA tubes and stored for up to 48 h at 4°C, to simulate short-term storage, could impact transcriptomic analysis, to see if leftover blood from standard hematological testing could be preserved and then used for this purpose.

## Materials and Methods

The study designs and collection procedures were explained to all participants who provided informed consent. The local ethics committee (long-term storage: SSCREC 2016–28; short-term storage: SSCREC17-32) approved the study designs, and all collection procedures were carried out according to the Helsinki Declaration. Blood samples were collected by trained phlebotomists.

### Experimental Design—Long-Term Storage

The long-term storage study was designed considering the following: 1) the ideal situation (RNA collection/extraction according to manufacturer instructions) as a reference and 2) the real situation that is faced by anti-doping biobanks, where whole blood samples are stored in tubes without an RNA stabilization reagent (e.g., K_2_EDTA tubes).

### Sample Collection and Storage

Whole blood was collected from an antecubital vein using a closed vacuette system from 12 male nonathletes (age: 23.9 ± 3.2; weight (kg): 80.0 ± 13.4; body fat percentage: 12.5 ± 2.9). A 3 mL whole blood sample was collected into a Tempus™ Blood RNA Tube (Life Technologies, Carlsbad, CA, United States), and a 10-mL whole blood sample was collected into a BD® EDTA tube (K_2_EDTA, Plymouth, United Kingdom). Immediately after collection, the Tempus™ tubes were vigorously shaken for 10 s and incubated at room temperature (RT) for ∼3 h and then stored at −80°C before subsequent analysis. After collection, the K_2_EDTA tube was gently inverted 10 times, incubated at 4°C for 1–4 h and then stored at −80°C for further analyses. During the 4°C incubation period for the K_2_EDTA tube, 1.25 mL of blood was pipetted into aliquots of 250 µL into five separate 1.5 mL TubeOne® Microcentrifuge tubes (Starlab, Milton Keynes, United Kingdom). One of these aliquots was used for RNA extraction from a “fresh” blood sample, and the other four tubes were immediately stored at −80°C.

### RNA Extraction

**FIGURE 1:**
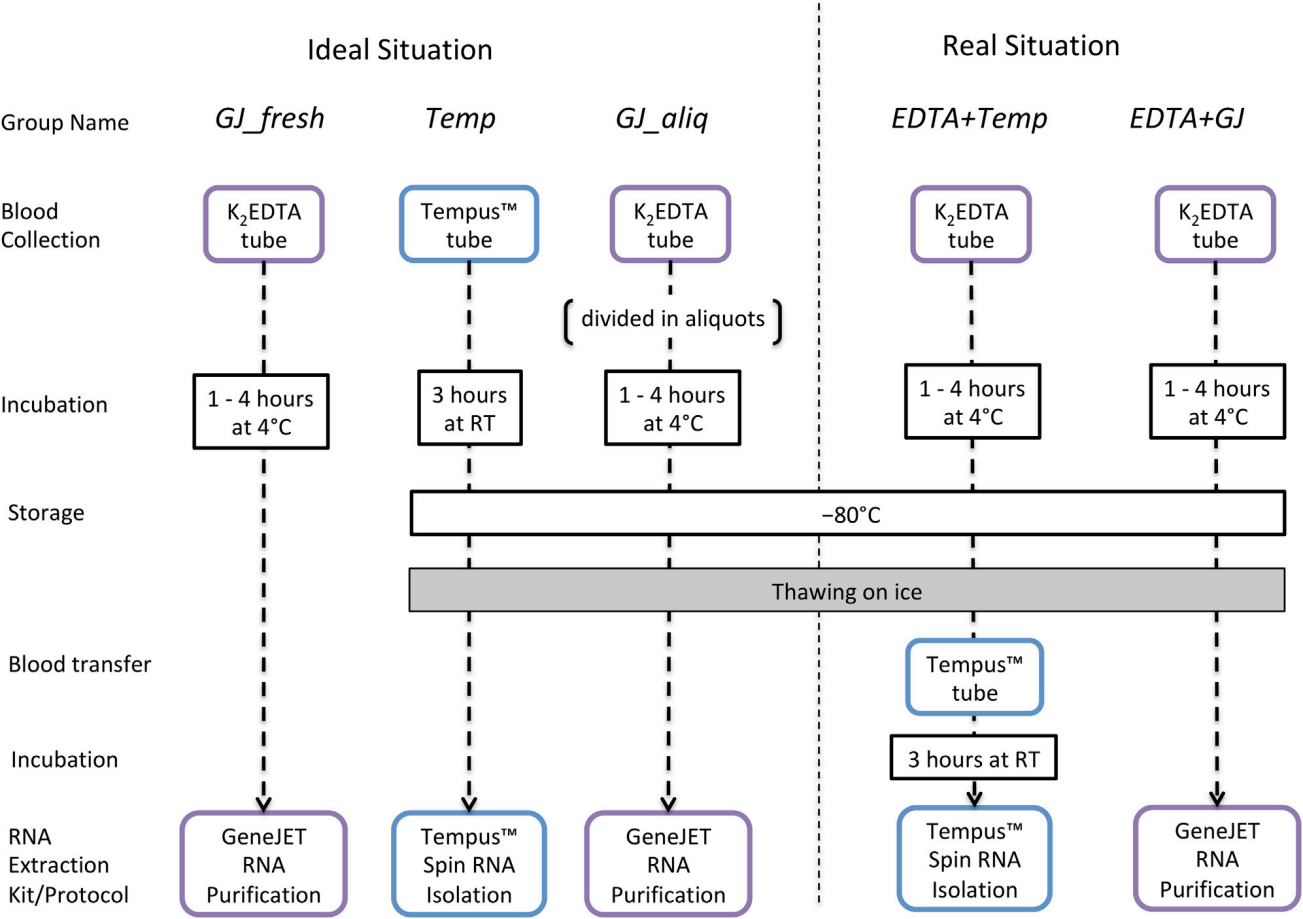
Long-term storage study design to evaluate RNA integrity and quality of samples collected in K_2_EDTA or Tempus™ blood tubes that have been kept at −80°C. **Ideal situation:** *GJ_fresh:* collected using a K_2_EDTA tube, incubated at 4°C (for up to 4 h), and extracted using the GeneJET protocol; *Temp*: collected/stored/extracted following the standard Tempus™ protocol; *GJ_aliq*: collected using a K_2_EDTA tube; a 250-µL aliquot is taken and stored in a 1.5-mL Microcentrifuge tube (according to manufacturer instructions) and then thawed with 250 µl of kit buffer and extracted using the GeneJET protocol. **Real situation:** *EDTA + Temp:* collected/stored using a K_2_EDTA tube, stored at −80°C, and thawed on ice; then 3 mL of whole blood was pipetted into a Tempus™ tube, vigorously shaken for 10 s, incubated at RT (∼3 h), and then extracted using the Tempus protocol; *EDTA + GJ:* collected/stored using a K_2_EDTA tube, stored at –80°C, and thawed on ice; next, 250 µl of whole blood was pipetted into a 1.5-mL Microcentrifuge tube and then extracted using the GeneJET protocol.

Total RNA was extracted from whole blood using two protocols: 1) the Tempus™ Spin RNA Isolation Kit (Life Technologies, Carlsbad, CA, United States) and 2) the GeneJET RNA Purification Kit (Thermo Fisher Scientific, Vilnius, Lithuania) following manufacturer instructions (User Guide, 2019a; User Guide, 2019b). The Tempus™ kit was chosen because it has been used in our previous studies (Durussel et al., 2016; Wang et al., 2017a) and as such facilitates comparability with previous results. The GeneJET kit was chosen because it is specific to extracting RNA from low volumes of frozen blood. In addition, both protocols were adapted for frozen blood samples that were collected/stored using K_2_EDTA tubes. The remaining blood sample inside the 10 mL K_2_EDTA tube was thawed on ice for ∼1.5 h, and then 3 mL of whole blood was used to perform the Tempus™ protocol and 250 µL was used for the GeneJET protocol. Considering the manufacturer and adapted protocols, RNA extraction was performed using five different protocol designs (Figure 1). After extraction, all RNA samples were stored at −80°C until further analysis.

### RNA Quality and Purity Analysis

RNA quantity and purity were assessed using a Nanodrop® ND-2000 Spectrophotometer (Thermo Fisher Scientific, Waltham, MA, United States). RNA integrity (RIN, RNA integrity number) was assessed using an Agilent® 2100 Bioanalyzer with an Agilent® RNA 6000 Nano Kit (Agilent Technologies, Santa Clara, CA, United States).

### RNA Microarray Analysis

RNA samples of 8 subjects from four different storage/extraction protocols (*Temp, EDTA + Temp, GJ_aliq,* and *EDTA + GJ*) with the highest quality were selected for the following analysis (*GJ_fresh* was not included because this did not simulate using frozen/biobanked samples nor was it used in previous studies with anti-doping purposes). One hundred nanograms of purified total RNA was processed using the Affymetrix GeneChip® Human Transcriptome 2.0 Array and a GeneChip® WT Plus Reagent Kit according to manufacturer instructions (Affymetrix, Thermo Fisher Scientific, Waltham, MA, United States). Transcriptome Analysis Console (TAC) 4.0 software (Life Technologies, Carlsbad, CA, United States) performed initial data QC, visualization of array scanning data, and transcript expression analysis. A false discovery ratio (FDR) of ≤0.05 and a fold change of >1.1 or <–1.1 were applied. The ideal situations were considered as reference groups.

### RNA-Sequencing

Seven RNA samples with the highest RIN values from each of the groups *Temp* and *EDTA + Temp* were selected for RNA-Seq analysis. rRNA was depleted from 200 ng of purified total RNA with RIN ≥7 using an MGIEasy rRNA Depletion Kit (BGI, Shenzhen, China). dsDNA libraries (with conditions for a 250-bp Insert Size) were created from the rRNA-depleted eluate using an MGIEasy RNA Directional Library Prep Set. dsDNA library quantity was assessed using a Thermo Fisher Scientific Qubit® dsDNA High Sensitivity Assay Kit and a Qubit® Fluorometer (Thermo Fisher Scientific, Waltham, MA, United States). The quality of the fragment size distribution of the dsDNA library was assessed by visual inspection of electropherograms created using an Agilent® DNA 1000 Kit on an Agilent® 2100 Bioanalyzer. Only dsDNA libraries with satisfactory fragment size distributions were carried forward onto the next steps, and dsDNA libraries were re-created for any samples with aberrant electropherograms or low concentrations. dsDNA libraries were circularized and converted into ssDNA libraries using an MGIEasy Circularization Kit. ssDNA library concentration was assessed using a Thermo Fisher Scientific Qubit® ssDNA Assay Kit and a Qubit® Fluorometer. DNA nanoballs (DNBs) were prepared from the ssDNA library pools, with a 40-fmol ssDNA library for each reaction, using an MGI DNBSEQ-G400RS High-throughput Sequencing Set (BGI, Shenzhen, China). DNB concentration was assessed using a Thermo Fisher Scientific Qubit® ssDNA Assay Kit and a Qubit® Fluorometer. DNB preparations >8 ng/μL were loaded onto two flow cells (containing equal numbers of samples) using an MGIDL-200H Portable DNB Loader (BGI, Shenzhen, China). The flow cells were placed on an MGI DNBSEQ-G400 sequencer (BGI, Shenzhen, China) and subjected to PE100 sequencing.

### RNA-Sequencing Gene Expression Analysis

Reads were mapped to Human Genome GRCh37 reference (hg19) using STAR 2.5.3. Gene counts were obtained using an annotation model based on RefSeq Transcripts (release 96) and normalized by fragments per kilobase million (FPKM) and by adding 0.0001 to all read counts, to allow statistical testing. Differential gene expression was determined by Partek Gene Set Analysis (GSA) using default settings. Genes presenting a fold change >1.1 or <–1.1 (a FC of 1.1) with *p* ≤ 0.05 for each comparison were considered differentially expressed.

### Experimental Design—Short-Term Storage

**FIGURE 2:**
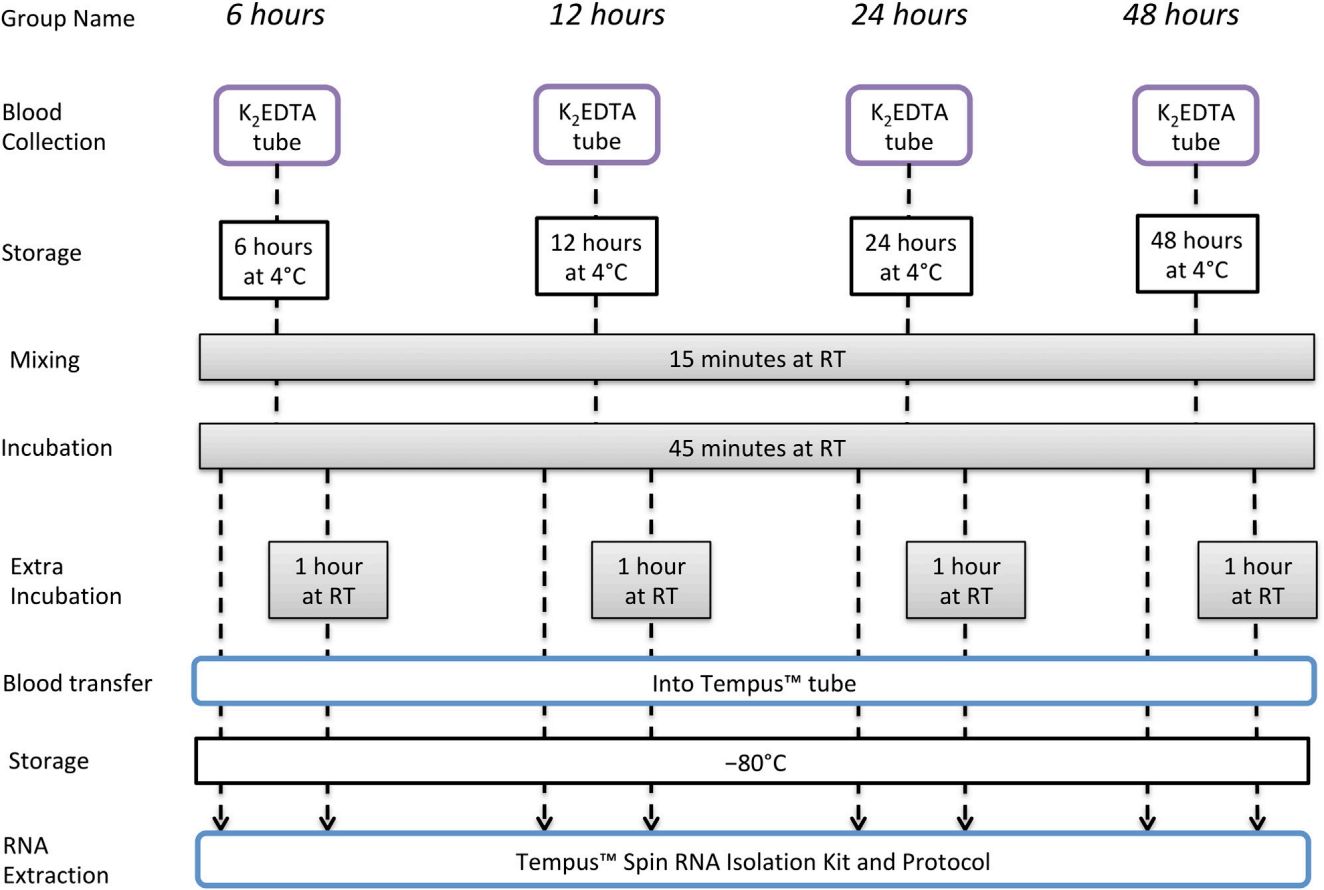
Short-term storage study design to evaluate RNA integrity and quality of samples collected and stored in K_2_EDTA tubes and then incubated at RT.

Only one participant from the long-term storage study also took part in the short-term storage study, and sampling was separated by > 12 months. Whole blood was collected from an antecubital vein of 14 male nonathletes, using a closed vacuette system in four separate 3-mL K_2_EDTA tubes. After collection, each of the four tubes was stored at 4°C for 6, 12, 24, or 48 h. At each of these time points, one of the K_2_EDTA tubes was removed from 4°C and maintained at RT for a total of 60 min to simulate the time required for sample registration and analysis. For the first 15 min of these 60 min at RT, the tube was placed in a sample mixer, and for the remaining 45 min, the tube remained upright at RT. After 60 min, 1.5 mL of whole blood was then removed from the tube and added to a new tube containing 3 mL of Tempus™ Blood RNA preservative, and this tube was shaken vigorously for 10 s. After a final 2 h at RT, the remaining 1.5 mL of whole blood was added to 3 mL of Tempus™ Blood RNA stabilizing reagent in a different tube, and this was shaken vigorously for 10 s. This was to simulate an extended registration and analysis time as can potentially occur in big events with a very high number of samples. All samples were then stored at −80°C until RNA extraction. The experimental design is illustrated in Figure 2.

### RNA Extraction and Quantification

Total RNA was extracted from whole blood using a Tempus™ Spin RNA Isolation Kit (Life Technologies, Carlsbad, CA, United States) following manufacturer instructions (User Guide, 2019b). RNA quality and purity were assessed as described in the long-term storage study methods section.

### RNA-Sequencing

Four samples, with the highest RIN value from each of the four time points (6, 12, 24, and 48 h spent incubating at 4°C), which were all incubated at RT for 1 h prior to the addition of RNA preservative, were selected for RNA-Seq and analyzed as described above in the long-term storage study methods section.

### Statistical Analysis

Descriptive data were presented as mean values and standard deviation (SD). The difference of integrity and purity values between the groups was determined using a generalized linear model, corrected by Bonferroni *post hoc*. The analyses of RNA quantity and quality were performed using SPSS software (v.23) with alpha at *p* ≤ 0.05.

## Results

### Long-Term Storage

**FIGURE 3:**
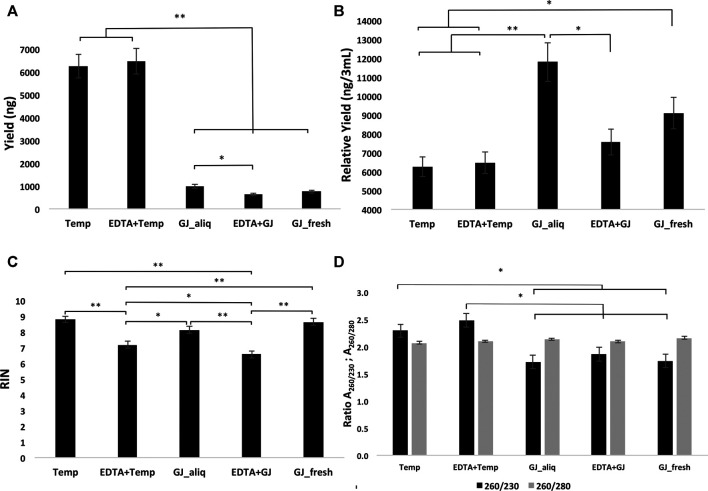
Yield **(A)** *,* relative yield **(B)** *,* RIN **(C),** and absorbance ratio **(D)** of RNA extracted from whole blood samples using the five different protocols. RIN, RNA integrity number; Temp, Tempus protocol; EDTA + Temp, K_2_EDTA tube plus Tempus protocol; GJ_aliq, GeneJET aliquoted protocol; EDTA + GJ, K_2_EDTA tube plus GeneJET protocol; GJ_fresh, Fresh blood and GeneJET protocol. The relative yield was reached by multiplying the final value of GeneJET protocol RNA extraction by 12 (as the starting volume is 12-fold lower than that of the Tempus protocol); * = *p* ≤ 0.05; ** = *p* ≤ 0.001. The graphs show mean values ±SD.

The average RNA yield of the Tempus™ extraction kit was higher than that of the GeneJET kit (Figure 3A). There were no statistical differences for the mean RIN of groups within the ideal situation. The ideal situation groups had a significantly higher mean RIN than the real situation groups. In addition, *EDTA + Temp* had a statistically significant higher mean RIN than *EDTA + GJ* (Figure 3C). RNA purity was analyzed using two absorbance ratios (A_260/280_ and A_260/230_). All groups demonstrated A_260/280_ > 2, with statistical differences between *Temp* and *GJ_fresh* (Figure 3D). Therefore, there was no significant difference in the comparison of A_260/230_ between the Tempus™ and GeneJET kits, although this difference was statistically significant in the inter-group comparisons (Figure 3D) (see values of the mean and comparison in Supplementary Material S1).

**FIGURE 4:**
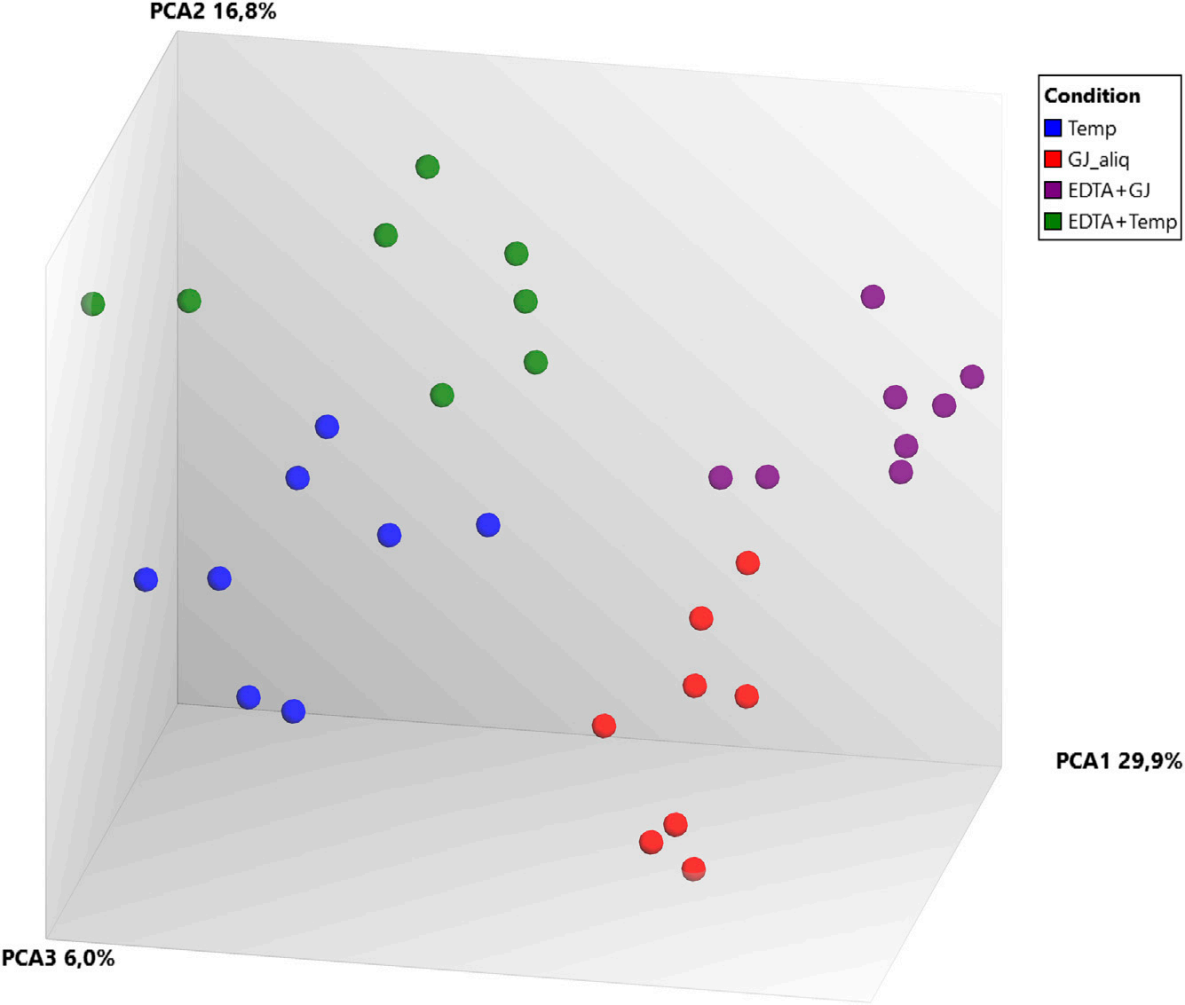
Microarray gene expression analysis. PCA, principal component analysis; Temp, Tempus protocol; EDTA + Temp, K_2_EDTA tube plus Tempus protocol; GJ_aliq, GeneJET aliquoted protocol; EDTA + GJ, K_2_EDTA tube plus GeneJET protocol.

Principal component analysis (PCA) (Figure 4) of microarray data demonstrated a separation between ideal situation groups and real situation groups and a lower difference intra-kit (*EDTA + Temp* vs. *Temp* and *EDTA + GJ* vs. *GJ_aliq*) than inter-kit (*GJ_aliq* vs. *Temp*). The comparison between *EDTA + Temp* vs. *Temp* showed the lowest number of differentially expressed transcripts (*n* = 4,058, which means 6.01% of the 67,528 coding and non-coding transcripts analyzed using the microarray), while *EDTA + GJ* vs. *GJ_aliq* demonstrated 6,324 (9.37%) and *GJ_aliq* vs. *Temp* revealed 13,678 (20.26%) differentially expressed transcripts.

**FIGURE 5:**
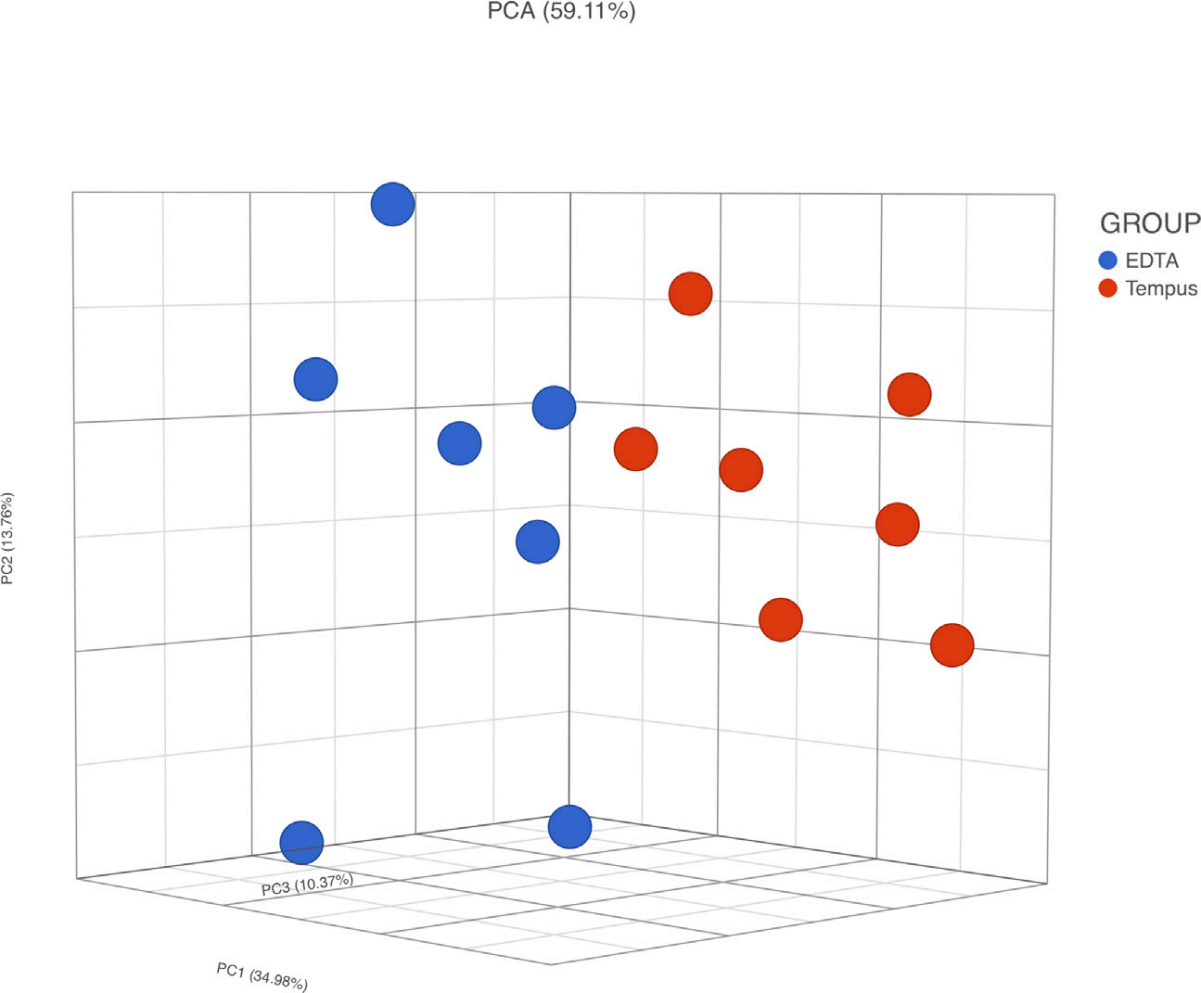
RNA-Seq gene expression analysis. PCA, principal component analysis; Temp, Tempus protocol; EDTA + Temp, K_2_EDTA tube plus Tempus protocol.

The PCA of RNA-Seq data comparing *Temp* and *EDTA + Temp* also showed two clusters, according to the sampling tube (Figure 5). The gene expression analysis showed that 658 genes were differentially expressed (which means 3.4% of mapped genes), with 269 being upregulated and 389 downregulated (list of differentially expressed transcripts in Supplementary Material S2). None of the transcripts described in previous studies relating to rHuEPO doping (Durussel et al., 2016; Wang et al., 2017a) were differently expressed.

### Short-Term Storage Results

**FIGURE 6:**
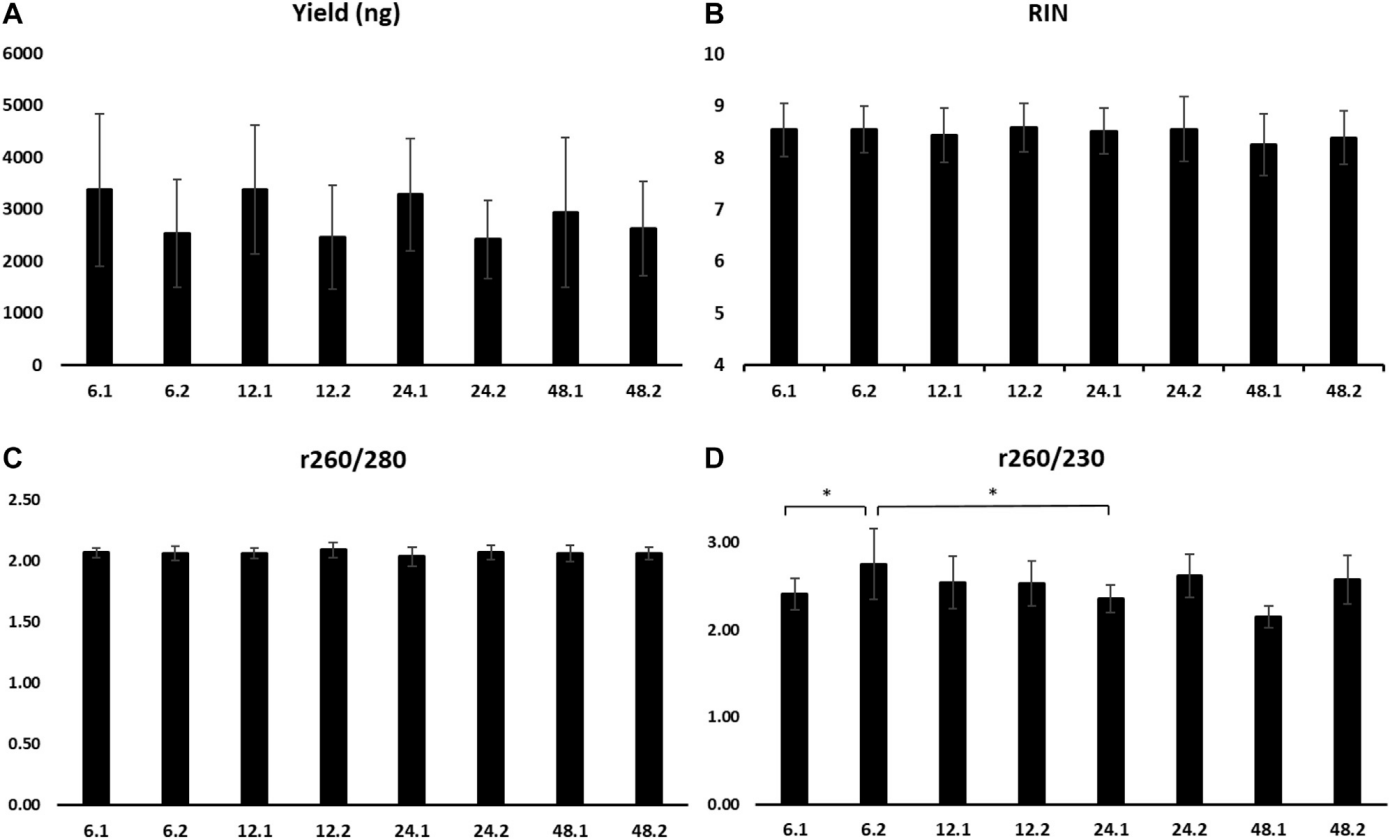
Yield **(A)** *,* RIN **(B)** *,* absorbance ratio _260/280_ **(C),** and absorbance ratio _260/230_ **(D)** of RNA extracted from samples stored at 4°C for 6, 12, 24, and 48 h, followed by 1 h (0.1) or 2 h (0.2) at room temperature. RIN, RNA integrity number.

There were no statistical differences for the mean RIN and yield amongst all eight time points (Figures 6A,B). When comparing all samples that were incubated at RT for 1 or 2 h, the RNA yield of 1 h incubation was significantly higher (3,539.8 ± 1,291.2 vs. 2,506.3 ± 911.5 ng, *p* ≤ 0.05).

A_260/280_ did not present differences among time points (Figure 6C). A_260/230_ revealed a significantly higher mean of the time points 6 h at 4°C and 2 h at RT compared to 6 and 24 h at 4°C and 1 h at RT (2.75 ± 0.4 vs. 2.41 ± 0.18 and 2.35 ± 0.16, *p* ≤ 0.05, respectively) (Figure 6D).

**FIGURE 7:**
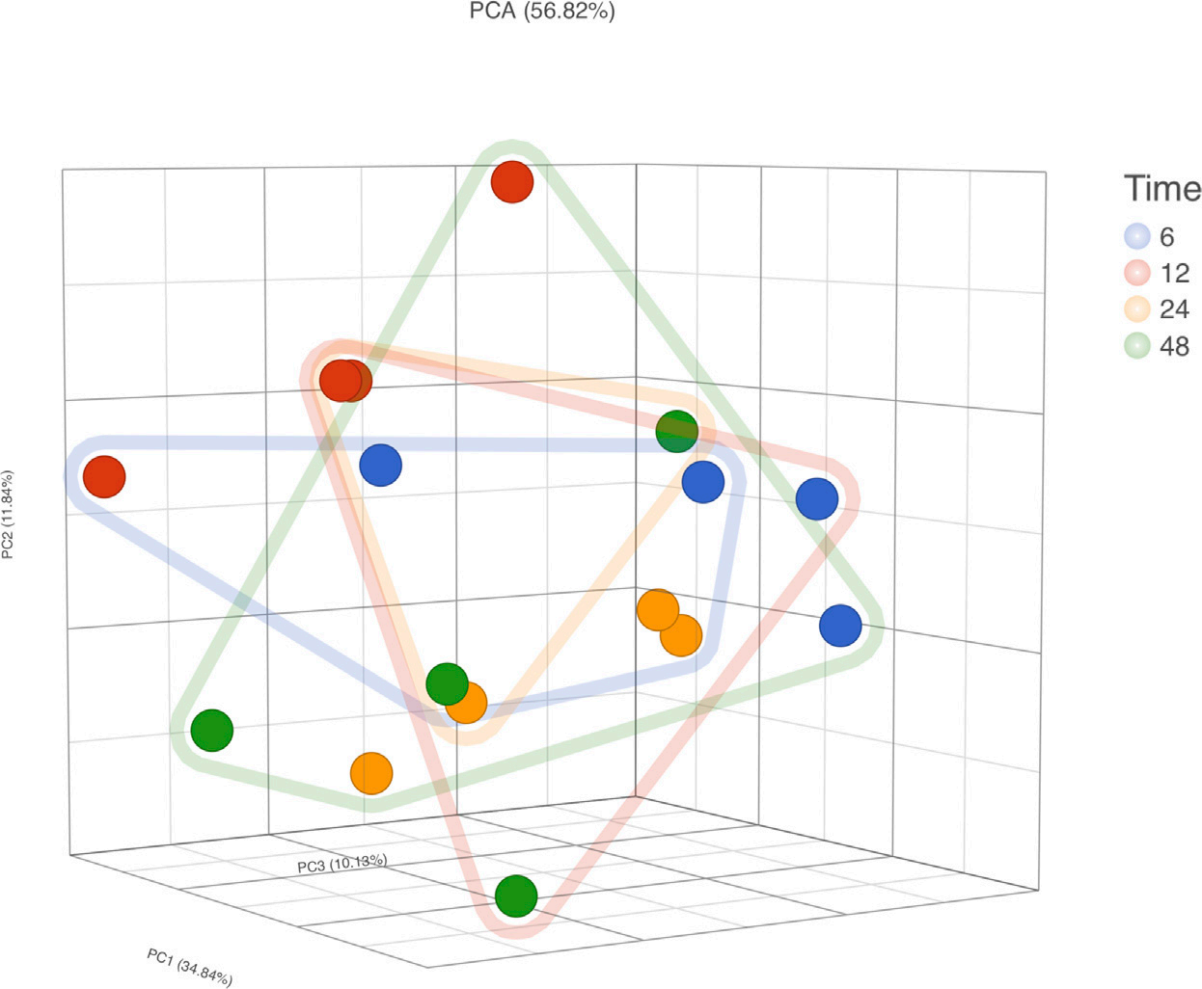
RNA-Seq gene expression analysis. PCA, principal component analysis; Dot Colors, sample ID; Frame lines, time.

RNA-Seq was conducted on four participants’ samples, with each sample having a 1 h RT incubation at each time point (i.e., 6, 12, 24, and 48 h; *n* = 16 samples in total). PCA did not demonstrate clear clusters regarding time of storage but did show clusters regarding samples of the same subjects (Figure 7). With 6 h storage as a reference group, the number of differentially expressed genes was 19, 45, and 70 in comparison to 12, 24, and 48 h, respectively (which means 0.37, 0.88, and 1.37% of mapped genes). Of the 19 differentially expressed genes in the comparison of 6 vs. 12 h, 9 overlapped with the 45 in the comparison of 12 vs. 24 h. Furthermore, 40 of those 45 overlapped with the 70 differentially expressed in the comparison of 6 vs. 48 h (list of differentially expressed genes in Supplementary Material S3). Again, none of the transcripts described in previous studies relating to rHuEPO doping (Durussel et al., 2016; Wang et al., 2017a) were differently expressed.

## Discussion

PED usage can create a gene expression signature, and transcriptomic analysis of these signatures can be a powerful tool to improve the anti-doping testing system (Reichel, 2011; Pitsiladis et al., 2014; Wang et al., 2017b). However, there are many requirements for the implementation and validation of this method to consider it for the current anti-doping system. RNA quality and quantity are of paramount importance to accurate gene expression analyses. Therefore, the choice of extraction protocol is a crucial step for downstream outcomes. Currently, anti-doping blood samples are not collected into tubes that contain RNA preservatives, whereas an appropriate RNA collection blood tube was used in rHuEPO gene expression discovery studies (Durussel et al., 2016; Wang et al., 2017a). This present study aimed to design a protocol to enable the comparison of current anti-doping sampling and storage methods with previous anti-doping gene expression signature studies and, additionally, see if long-term stored anti-doping samples could potentially be used for gene expression analysis.

In the long-term storage study, the results showed that both methods (i.e., Tempus™ and GeneJET) extracted sufficient RNA quantities from blood collected in a K_2_EDTA tube and stored at −80°C. The standard Tempus™ protocol provided noticeably higher yield (Figure 3A); however, it does not mean better efficiency, due to the lower starting sample volume of the GeneJET protocol (3 mL vs. 250 µL). Both Tempus™ and GeneJET extraction kits produced RNA with a mean absorbance ratio (A_260/280_) > 2 (Figure 3D), which is considered “pure” for further RNA applications (NanoDrop, 2009). Although *GJ_fresh* had a statistically higher mean than *Temp*, in practice, this difference might not be relevant as both values are sufficiently high. A_260/230_ can indicate the presence of co-purified contaminants (such as carbohydrates and salts) if this ratio presents a value lower than 1.8 (Lee et al., 2014). Samples extracted using a Tempus™ kit showed mean values higher than 2.2 while GeneJET extraction demonstrated statistically lower values (on average, ≤1.8). Although the Tempus™ kit did show a better result for this parameter, the impact of this difference on downstream analyses is not clear.

RNA integrity was evaluated according to the RIN value (numeric scale from 1 to 10, where 1 means the most degraded and 10 means the most intact profile (Mueller et al., 2004)). The RIN value demonstrated that ideal situation groups (following manufacturer instructions) had significantly higher mean values than the real situation groups (adapted protocol) (Figure 3C). In the adapted protocol, the blood samples inside K_2_EDTA tubes were thawed on ice for ∼1.5 h, followed by the RNA extraction protocol. This thawing time could affect RNA integrity as mRNA structures could be degraded by RNase enzymes (Swift et al., 2000). Nevertheless, the average RIN values were considered acceptable (Fleige and Pfaffl, 2006; Beekman et al., 2009). Of note is that a PCA plot of microarray data (Figure 4) shows that expression profiles are more similar for samples that are extracted using the same manufacturer kit. This result can also be observed for the number of differentially expressed transcripts when *EDTA + Temp* and *Temp* were compared. A PCA plot generated by RNA-Seq analysis also discriminated between the *Temp* and *EDTA + Temp* clusters, as well as 658 genes differentially expressed. Considering the application of transcriptomic-based technologies in order to improve the efficacy of doping detection, we analyzed 49 target and five housekeeping (HK) genes described in previous studies (Wang et al., 2017a) (list of genes in Supplementary Material S4). None of the target and HK genes were differentially expressed in the comparison of *EDTA + Temp* vs. *Temp*, which could suggest that target genes and their controls were not impacted by the freezing/thawing process and could be used as doping biomarkers in biobanked samples. Some of these genes (*ALAS2*, *CA1*, and *SLC4A1*) have already been shown to be promising blood doping biomarkers even when using blood samples collected by Dried Blood Spot (DBS) technologies (Salamin et al., 2019; Loria et al., 2020)”. The expression of blood transcriptomic biomarkers remains significantly different after 3 days using DBS blood samples (Loria et al., 2020) and up to 3 weeks after using whole blood collected using Tempus^TM^ tubes (Wang et al., 2017a). *ALAS2* is a tissue-specific gene and plays an important role in heme biosynthesis, providing instructions for the expression of an enzyme called 5′-aminolevulinate synthase 2 or erythroid ALA-synthase, found only in erythroblasts. Therefore, *ALAS2* is mediated by erythroid-specific factors and has an impact on hemoglobin formation (Ajioka et al., 2006; Wang et al., 2017a). Carbonic anhydrases (CAs) are metalloenzymes that catalyze the important interconversion between CO_2_ and bicarbonate and are involved in many physiological and pathological processes such as respiration and CO_2_ transport, pH and CO_2_ homeostasis, biosynthetic reactions, calcification, and tumor progression (Kupriyanova et al., 2017). *SLC4A1* encodes a protein member of the Solute Carrier 4 family of bicarbonate transporters (SLC4A1), which is expressed in the erythrocyte membrane, involved in the transport of carbon dioxide from tissues to the lungs (Reithmeier et al., 2016).

**FIGURE 8:**
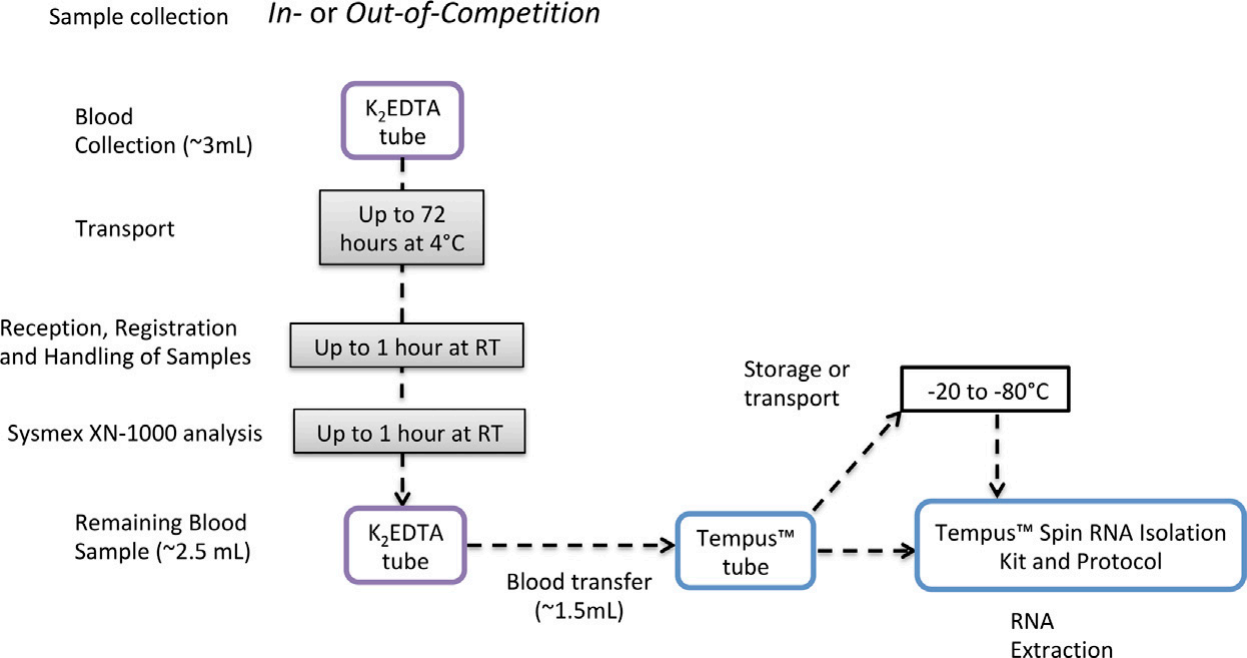
Proposed pipeline for integration of gene expression analysis into the current anti-doping collection protocols, using Tempus^TM^ tubes to preserve RNA integrity.

The short-term storage experiments were designed based on the observed logistics of sample collection, storage, transport, registration, and analysis of anti-doping samples during anti-doping testing performed at the IAAF World Championships 2019 in Doha, Qatar. WADA Code guidelines describe that for the analysis of *Prohibited Substances* and *Methods*, two blood samples should be collected from an athlete (samples A and B) in two 3-mL K_2_EDTA tubes (WADA, 2021c). Blood samples are dispatched as soon as possible after collection to an anti-doping laboratory and analyzed within 72 h from collection (WADA, 2021c). During the IAAF World Championships 2019, all samples were analyzed between 6 and 24 h after collection. WADA guidelines also require a sample to be mixed thoroughly for a minimum period of 15 min at room temperature using an appropriate mixer (e.g., roller mixer) prior to analysis once the tube is removed from the tamper-proof container. Recently, the Sysmex XN-1000 automated hematology analyzer has been introduced as the hematology analyzer of choice for all WADA-accredited and approved laboratories (WADA, 2021b; XN, 2021). Each blood sample analyzed using the Sysmex XN-1000 must be analyzed twice, and the results of the second sample are used to confirm those of the first; if the difference between both is above the accepted criteria, the analysis is repeated. Due to that process, blood samples can remain at RT for 1 or 2 h. The Sysmex XN-1000 aspirates 88 µL of whole blood for each single analysis (XN, 2021), and once the duplicate result is within the limits, the result is accepted (WADA, 2021a). After this analysis, the remaining volume of whole blood could be used for gene expression analysis as shown in Figure 8. To fit in this context, the short-term storage experiment was designed to investigate if the storage protocol that these remaining whole blood samples collected into K_2_EDTA tubes would be subjected to would impact RNA quality, purity, integrity, and transcriptomic analysis. Unexpectedly, the RIN value of the samples was not significantly different between all the time points, and neither was the RNA yield. However, comparing all samples at RT for 1 h vs. 2 h, the yield was significantly lower in 2 h groups but still sufficiently high for RNA-Seq. RNA-Seq analysis demonstrated that the number of differentially expressed genes increases the longer the sample is stored at 4°C prior to the addition of RNA preservative. However, none of the gene targets described in the gene expression signature of rHuEPO administration (Wang et al., 2017a) (list of genes in Supplementary Material S4) presented different expression in the short-term storage study. The low number of transcripts differently expressed across time of storage, including the absence of blood doping transcript targets, supports the possible integration of gene expression collection procedures and analytical methods into samples left over from routine hematological anti-doping testing.

The short-term storage experiment showed that 1.5 mL of whole blood collected in K_2_EDTA, simulating remaining blood from standard hematological testing, could be used for extracting RNA that has sufficient yield, quality, and integrity for gene expression analyses. The long-term storage experiment also demonstrated that 3 mL whole blood collected in K_2_EDTA tubes and stored at −80°C can be used for gene expression analysis. In both of the studies, none of the gene targets described in the gene expression signature of rHuEPO administration (Wang et al., 2017a) presented different expression. In summary, despite many challenges, the results of this study are very encouraging and reveal that RNA yield and purity are not compromised and that significant progress has been made toward how to integrate these new transcriptomic tests into the current anti-doping testing system, including using samples remaining from standard hematological testing. The limitations of the present study include the use of a nonathlete population, and although the sample collection procedures tried to simulate the International Standard for Laboratories (ISL) protocols, the samples were not collected in a real anti-doping testing situation. Future studies with a higher number of participants/samples, including known doped blood samples in the aforementioned situations, can give more support to the validation of the protocols designed in the present study.

## Data Availability

The data presented in the study are deposited in the Gene Expression Omnibus (GEO) repository, accession numbers; Microarray: GSE181970 and RNA-Seq (unified): GSE183133, or in these links: microarray: https://www.ncbi.nlm.nih.gov/geo/query/acc.cgi?acc=GSE181970, RNA-Seq: https://www.ncbi.nlm.nih.gov/geo/query/acc.cgi?acc=GSE183133.
