# Supplementary material for: Integrating Whole Blood Transcriptomic Collection Procedures Into the Current Anti-Doping Testing System, Including Long-Term Storage and Re-Testing of Anti-Doping Samples
Source: Front Mol Biosci. 2021 Oct 26;8:728273. doi: 10.3389/fmolb.2021.728273 (PMC8576497; doi:10.3389/fmolb.2021.728273)
Supplement: Supplementary file 4 [file Table4.docx]

**Supplementary File Data 4.**

Previously described in:

Wang G, Durussel J, Shurlock J, et al. Validation of whole-blood transcriptome signature during microdose recombinant human erythropoietin (rHuEpo) administration. *BMC Genomics*. 2017;18(Suppl 8). doi:10.1186/s12864-017-4191-7

List of target genes previously investigated:

| **Gene Symbol** | **Gene Name** |
| --- | --- |
| **ACTB** | Actin, beta |
| **ACTR10** | Actin-related protein 10 homolog (S. cerevisiae) |
| **ADIPOR1** | Adiponectin receptor 1 |
| **ALAS2** | 5'-aminolevulinate synthase 2 |
| **BCL2L1** | BCL2-like 1 |
| **BPGM** | 2,3-bisphosphoglycerate mutase |
| **C1ORF128** | chromosome 1 open reading frame 128 |
| **CA1** | Carbonic anhydrase I |
| **CCR7** | Chemokine (C-C motif) receptor 7 |
| **CD247** | CD247 molecule |
| **CD3D** | CD3d molecule, delta (CD3-TCR complex) |
| **CSDA** | Cold shock domain protein A |
| **DCAF12** | DDB1 and CUL4 associated factor 12 |
| **E2F2** | E2F transcription factor 2 |
| **EEF1D** | Eukaryotic translation elongation factor 1 delta (guanine nucleotide exchange protein) |
| **EPB42** | Erythrocyte membrane protein band 4.2 |
| **FAM46C** | Family with sequence similarity 46, member C |
| **FBXO7** | F-box protein 7 |
| **FECH** | Ferrochelatase |
| **GMPR** | Guanosine monophosphate reductase |
| **GUK1** | Guanylate kinase 1 |
| **GYPB** | glycophorin B |
| **GYPE** | Glycophorin E (MNS blood group) |
| **HBD** | Hemoglobin, delta |
| **HBE1** | Hemoglobin, epsilon 1 |
| **IFI27** | interferon, alpha-inducible protein 27 |
| **KRT1** | Keratin 1 |
| **LEF1** | Lymphoid enhancer-binding factor 1 |
| **MARCH8** | membrane-associated ring finger (C3HC4) 8 |
| **MIF** | Macrophage migration inhibitory factor (glycosylation-inhibiting factor) |
| **OSBP2** | Oxysterol binding protein 2 |
| **PITHD1** | PITH (C-terminal proteasome-interacting domain of thioredoxin-like) domain containing 1 |
| **RAP1GAP** | RAP1 GTPase activating protein |
| **RBM38** | RNA binding motif protein 38 |
| **RNF213** | Ring finger protein 213 |
| **ROPN1B** | Ropporin, rhophilin associated protein 1B |
| **SELENBP1** | Selenium binding protein 1 |
| **SERPINA13** | Serpin peptidase inhibitor, clade A (alpha-1 antiproteinase, antitrypsin), member 13 (pseudogene) |
| **SIAH2** | seven in absentia homolog 2 |
| **SGK223** | Homolog of rat pragma of Rnd2 |
| **SKAP1** | Src kinase associated phosphoprotein 1 |
| **SLC4A1** | Solute carrier family 4, anion exchanger, member 1 (erythrocyte membrane protein band 3, Diego blood group) |
| **SLC6A10P** | Solute carrier family 6 (neurotransmitter transporter, creatine), member 10 (pseudogene) |
| **SNCA** | Synuclein, alpha (non A4 component of amyloid precursor) |
| **STRADB** | STE20-related kinase adaptor beta |
| **TMOD1** | Tropomodulin 1 |
| **TNS1** | Tensin 1 |
| **TPRA1** | Transmembrane protein, adipocyte asscociated 1 |
| **TRIM58** | Tripartite motif-containing 58 |
| **UBXN6** | UBX domain protein 6 |
| **VEGFB** | Vascular endothelial growth factor B |
| **WDR40A** | WD repeat domain 40A |
| **XK** | X-linked Kx blood group |
| **YOD1** | YOD1 deubiquitinase |

List of target housekeeping genes used:

| **Gene Symbol** | **Gene Name** |
| --- | --- |
| **ACTB** | Actin, beta |
| **ACTR10** | Actin-related protein 10 homolog (S. cerevisiae) |
| **MRFAP1** | Mof4 family associated protein 1 |
| **PPIB** | Peptidylprolyl isomerase B (cyclophilin B) |
| **RAB11A** | RAB11A, member RAS oncogene family |
